# Inflammatory biomarkers and cardiac injury in COVID-19 patients

**DOI:** 10.3389/fpubh.2022.1024535

**Published:** 2022-11-24

**Authors:** Ayesha Mumtaz, Erum Rehman, Mohammad Anisur Rahaman, Shazia Rehman

**Affiliations:** ^1^School of Public Administration, Hangzhou Normal University, Hangzhou, China; ^2^Department of Mathematics, Nazarbayev University, Nur-Sultan, Kazakhstan; ^3^College of Public Administration, Zhejiang University, Hangzhou, China; ^4^Department of Sociology, Bangabandhu Sheikh Mujibur Rahman Science and Technology University, Gopalganj, Bangladesh; ^5^Department of Biomedical Sciences, Pak-Austria Fachhochschule, Institute of Applied Sciences and Technology, Haripur, Pakistan

**Keywords:** SARS-CoV-2, COVID-19, cardiac injury, severe patients, cardiac biomarkers, inflammatory biomarkers, coagulation biomarkers, mortality

## Abstract

**Introduction:**

Cardiac injury has received considerable attention due to the higher risk of morbidity and mortality associated with coronavirus disease. However, in a developing country, there is a scarcity of data on cardiac injury in COVID-19 patients related to inflammatory biomarkers.

**Methods:**

Therefore, the present research retrospectively analyzes data from three territorial hospitals in Pakistan's Punjab province to investigate the potential impact of the cardiac injury on the mortality and severity of COVID-19-infected patients. We evaluated 2,051 patients between January 16 and April 18, 2022, with confirmed COVID-19. The in-hospital mortality recorded for the selected sample size was about 16.28%.

**Results:**

The majority of the participants were identified as male (64%) with a median age of 65 years. Also, fever, fatigue, and dyspnea were reported as common symptoms. An aggregate of 623 patients (30.38%) had a cardiac injury, and when these patients are compared to those without cardiac injury, the participants were significantly older and had more comorbidities with higher leukocyte counts, elevated levels of C-reactive protein, interleukin-6, procalcitonin, myohemoglobin, creatinine kinase-myocardial band, serum creatinine, high-sensitivity troponin-I, N-terminal pro-B-type natriuretic peptide had a significant amount of multiple ground-glass opacity and bilateral pulmonary infiltration in radiographic results. Participants with heart injury required more non-invasive or invasive mechanical respiration than those who did not have a cardiac injury. Individuals with cardiac injury had higher rates of sepsis, acute respiratory distress syndrome (ARDS), d-dimer concentration, and respiratory failure than those without cardiac injury. Patients who had had a cardiac injury died at a higher rate than those who had not suffered cardiac damage. In the multivariable logistic regression analysis, participants with cardiac injury showed greater odds of COVID-19 mortality and were found associated with older age (OR = 1.99, 95% CI = 0.04–3.19), elevated cardiac troponin I (OR = 18.64, 95% CI = 13.16–23.01), the complication of sepsis (OR = 10.39, 95% CI = 7.41–13.39) and ARDS (OR = 6.65, 95% CI = 4.04–8.91).

**Conclusion:**

Cardiac injury is a frequent complication among patients with coronavirus-induced infection in Punjab, Pakistan, and it is significantly linked to a greater risk of in-hospital mortality.

## Introduction

Millions of people who have contracted the coronavirus disease 2019 (COVID-19), spurred by severe acute respiratory syndrome coronavirus 2 (SARS-CoV-2) (1), have experienced significant morbidity and fatality. The clinical manifestations of patients infected by COVID-19 have previously been identified by a growing body of research (2–4). The cumulative COVID-19 mortality rate was estimated to be about 2.3% among general laboratory-confirmed cases (5), while it exceeded ten percent in hospitalized settings and eventually approached 40% in severe patients (6). The pathogens are single-stranded RNA viruses that have a strong capability for rapid transformation and recombination. They can infect both people and animals through their respiratory system or gastrointestinal tract (7). SARS-CoV-2 infection develops as a consequence of the virus's surface S-protein interacting with angiotensin-converting enzyme 2 (ACE2), which functions as a viral sensor. ACE2 is predominantly found in the lungs and appears to be the virus's primary access point. It is also abundant in the heart, which might contribute to cardiac disorders (7, 8). The major therapeutic diagnoses against COVID-19 infection involve respiratory disorders, ranging from mild to potentially lethal acute respiratory distress syndrome (ARDS). Besides, as in other respiratory infections, pre-existing cardiovascular diseases and risk factors can increase the severity of COVID-19, leading to the aggravation and decompensation of chronic underlying cardiac pathologies as well as acute-onset of new cardiac complications (5), highlighting that myocardial injury can be present in approximately 12% of hospitalized patients with SARS-CoV-2 infection (9).

Cardiac injury has received considerable attention due to the higher risk of morbidity and mortality associated with coronavirus disease (10). Several mechanisms and patterns of injury have been linked to cardiovascular involvement in individuals with coronavirus disease (COVID-19) (11, 12). More than 30% of hospitalized patients with COVID-19 had cardiac involvement, as evidenced by increased troponin levels, which are linked to poor short-term outcomes (13–15). Elevated troponins, with or without a prior history of CVD, were linked to malignant arrhythmias, sudden respiratory failure, and increased mortality in a case series of 187 individuals with COVID-19 (3, 15). Surprisingly, individuals with established CVD but no increased troponins had a better prognosis. Acute cardiac injury signifies a poor prognosis, an urgent requirement for mechanical ventilation, and a higher rate of mortality. There is a scarcity of data on cardiac injury in COVID-19 patients in a developing country like Pakistan. Therefore, the present research retrospectively analyzes data from three territorial hospitals in Pakistan's Punjab province to investigate the potential impact of the cardiac injury on the mortality and severity of COVID-19-infected patients.

## Materials and methods

### Data source and study participants

All enrolled 2,763 patients, who were referred to the Benazir Bhutto Hospital of Rawalpindi, the Services Hospital of Lahore, and the Nishtar Hospital of Multan in the Punjab province from January 16, 2022, to April 18, 2022, were retrospectively and consecutively analyzed. As of April 29, 2022, the clinical results of the entire hospitalized patient population were obtained. All cases with SARS-CoV-2 infection in the present investigation have been identified grounded on interim direction from the WHO. According to the arrangements by the Pakistani government, all individuals identified with COVID-19 were treated at these three authorized hospitals. Written or oral informed consent was received from all participating patients and their families. The Ethics Review Committee of the chosen hospitals, which is a regulatory authority under the Ministry of Health of Pakistan, granted clearance for the study (KIIT 2022/PK 2022-104-MS 68).

**Figure 1:**
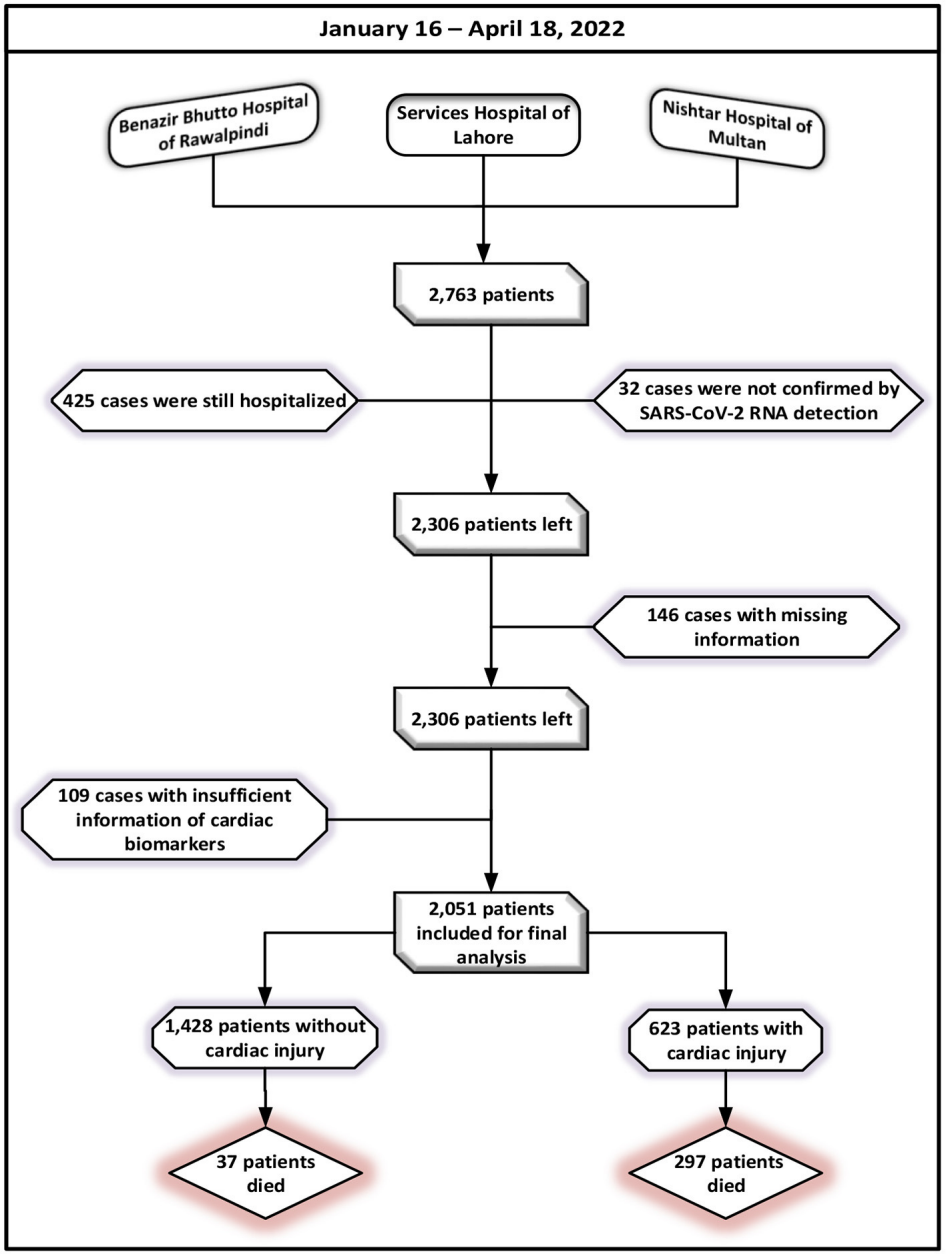
Patient recruitment flowchart.

Healthcare data from patient's medical records and attending doctors, including demographic attributes (gender, age), clinical findings on symptoms, co-morbid, laboratory parameters, imaging features, in-hospital therapies, complications, prognosis, and cardiac examination results (cardiac biomarkers, electrocardiography, and echocardiography). Any patient who had started experiencing an onset of fever, fatigue, cough, and/or body pains was considered in the study. Following the exclusion of patients who remained admitted to a hospital (*n* = 425) or were not identified by SARS-CoV-2 RNA detection (*n* = 32) as of April 18, 2022, a total of 2,306 eligible patients were left. In total of 2,160 participants were left for the analysis after additional exclusions for patients who did not have the most pertinent information in their medical records. The cases lacking cardiac biomarkers (*n* = 109), including creatinine kinase–myocardial band (CK-MB) and higher levels of troponin-I (hs-TNI) values, were also eliminated. After avoidance, a total of 2,051 cases with a confirmed diagnosis of SARS-CoV-2 have been cleared out for the final investigations. A flowchart for patient registration is shown in Figure 1.

The history of exposure has been reported as exposure to individuals who had been diagnosed with SARS-CoV-2. Serum cytokine levels (IL-6, TNF-α, and IL-8) were measured at the time of admission. It was determined that underlying co-morbidities existed following the International Classification of Diseases (revision 10). Cardiac biomarkers such as elevated troponin-I, CK-MB, and myohemoglobin were assessed at admission time. Chest radiography and computerized tomography (CT) were among the radiologic examinations. Patients were divided into groups based on whether or not they had suffered heart injury. Despite new abnormalities in electrocardiograms and echocardiograms, heart injury was characterized by elevated cardiac biomarkers in the blood (i.e., above the 99^th^ percentile upper reference limit). The Berlin concept was coined to characterize acute respiratory distress syndrome. The renal disease: Improving Global Outcome criteria were adopted to identify acute kidney damage.

Prior to final data input, participants' data were checked for consistency by two competent biostatisticians before being entered into an electronic database. In-hospital patient mortality, hospital discharge, and hospitalization were all included as clinical outcomes. On or before April 29, 2022, a follow-up was required. An acute COVID-19 incident was identified by the Infectious Diseases Society of America and the American Thoracic Society for Diagnosis and Treatment of Adults in Community-Acquired Pneumonia 2019.

### Detection of COVID-19

A confirmed COVID-19 infection has been defined as a real-time RT-PCR test result that is positive for nasal and oropharyngeal swab samples. All medical staff members who treat infectious patients have the professional training and expertise necessary to implement established infection prevention techniques and policies. All participants who presented in a single viral medium tube were asked to collect combined oropharyngeal and nasopharyngeal swabs collections while following transmission prevention measures. Swabs from the throat (oropharyngeal, OP) are extracted by immediately placing the swab into a sterile tube with 2 to 3 ml of viral transport media after swabbing the tonsils and posterior pharynx at least two to three rounds with a nylon-flocked swab while bypassing the tongue region. The biological samples were carefully secured and taken to the lab in strict accordance with the established methodology.

### Statistical analysis

For the chosen population, the fundamental clinical and epidemiological characteristics of patients with related co-morbidities, contraindications, and inpatient treatments have been compiled in percentages and frequencies. For continuous variables, the median and interquartile (IQ) ranges are descriptive statistics. Mann- Whitney U test was carried out for continuous data, while for continuous variables, the χ*2* test and Fisher exact tests were used as desired. We conducted a univariate study to investigate the factors linked to COVID-19 patient deaths. The multivariable logistic regression analysis, in which those factors were modified, was used to include the significant variables identified by the univariate analysis (*P* < *P* < 0.05). To report the relationship between patients with and without cardiac injury, an odds ratio (OR) with a 95 percent confidence interval (CI) was utilized. It has been shown that a *P* value of less than 5% is statistically significant. The SAS software, version 9.3 (SAS Institute Inc., Cary, NC, USA), was used for all statistical calculations unless otherwise stated.

## Results

### Baseline demographic and clinical characteristics of COVID-19 patients

Table 1 summarizes the patients' baseline demographic and clinical features. Of all 2,051 confirmed COVID-19 patients, 623 (30.4%) were with cardiac injury and 1,428 (69.6%) were without cardiac injury. The estimated median age of the study population was 65 years with an interquartile range of 53.0–78.0 years. The proportion of males (69%; 427 of 623) was observed higher among patients with cardiac injury as contrasted with those without cardiac injury. An aggregate of 1,130 patients was ≥65 years and approximately half of the patients (56%; 1,155 of 2,051) were recognized as smokers compared with non-smokers (35%; 221 of 623). Among patients with cardiac injury, about half of patients were pinned down as obese with a BMI ≥ 30. Almost 60% of cases in the study population showed up with an exposure history. COVID-19's first reported case was dated 26 February 2020. Approximately 10% of all the participated patients were detected as asymptomatic *via* computed tomography (CT) screening. Compared to cases without cardiac injury, patients with cardiac injury had to wait much longer from the time an infection started to the time they went to an outpatient facility and then to the time they were admitted. Among 2,051 patients, 39% of patients had hospital visits more than twice of whom 33% were cardiac injury cases.

**Table 1:** Baseline demographic and clinical characteristics of COVID-19 patients.

**Characteristics**	**All confirmed cases (%)**	**With cardiac injury** ***n* (%)**	**Without cardiac injury** ***n* (%)**	***P*-value**
**Age (years), median (IQR)**	65.0 (53.0–78.0)	72.0 (65.0–92.5)	62.0 (55.5–76.0)	<0.0001
<65	921 (45%)	180 (29%)	741 (52%)	0.051
≥ 65	1,130 (55%)	443 (71%)	687 (48%)	0.001
**Sex**				0.45
Male	1,313 (64%)	427 (69%)	886 (62%)	–
Female	738 (36%)	196 (31%)	542 (38%)	–
**Current smoking**				0.22
Yes	1,155 (56%)	402 (65%)	753 (53%)	–
No	896 (44%)	221 (35%)	675 (47%)	–
**BMI** **≥ 30 (Kg/m**^**2**^**)**	1,018 (50%)	332 (53%)	686 (48%)	0.031
**Exposure history**	1,236 (60%)	402 (65%)	834 (58%)	0.15
**Time from illness onset to outpatient visit (d), median (IQR)**	2 (1–5)	3 (1–6)	2 (1–4)	
**Time from illness onset to hospitalization (d), median (IQR)**	12 (8–15)	12 (9–16)	11 (8–14)	
**Number of hospital visits** **≥ 2**	801 (39%)	204 (33%)	597 (24%)	
**Signs & symptoms on arrival at the hospital**, ***n*** **(%)**				
Fever (temp ≥ 37 °C)	1,658 (81%)	468 (75%)	1,190 (83%)	0.013
Fatigue	1,420 (69%)	455 (73%)	965 (68%)	0.24
Dyspnea	1,109 (54%)	435 (70%)	674 (47%)	0.37
Sore throat	974 (47%)	310 (50%)	664 (46%)	0.53
Nasal congestion/rhinorrhea	926 (45%)	382 (61%)	544 (38%)	0.47
Dry cough	855 (42%)	192 (31%)	663 (46%)	0.11
Sputum production	777 (38%)	335 (54%)	442 (31%)	0.67
Chest pain	723 (35%)	332 (53%)	391 (27%)	0.13
Dizziness/headache	696 (34%)	202 (32%)	494 (35%)	0.79
Nausea and vomiting	554 (27%)	122 (20%)	432 (30%)	0.44
Chill	416 (20%)	235 (38%)	181 (13%)	0.18
Abdominal pain	366 (18%)	74 (12%)	292 (20%)	0.49
Diarrhea	327 (16%)	56 (9%)	271 (19%)	0.63
Myalgia or arthralgia	301 (15%)	72 (12%)	229 (16%)	0.55
**Co-existing conditions**, ***n*** **(%)**				
Hypertension	1,065 (52%)	537 (86%)	528 (37%)	<0.0001
DM	1,010 (49%)	512 (82%)	498 (35%)	0.001
Cerebrovascular diseases	986 (48%)	413 (66%)	573 (40%)	0.001
CHD	965 (47%)	403 (65%)	562 (39%)	0.001
COPD	521 (25%)	200 (32%)	321 (22%)	0.13
CHF	212 (10%)	101 (16%)	111 (8%)	<0.0001
Chronic renal disease	156 (8%)	66 (11%)	90 (6%)	0.39
Chronic liver disease	96 (5%)	36 (6%)	60 (4%)	0.24
Tuberculosis	88 (4%)	41 (7%)	47 (3%)	0.54
Asthma	55 (3%)	33 (5%)	22 (2%)	0.001
Hepatitis B	23 (1%)	17 (3%)	6 (<1%)	0.34
Carcinoma	10 (<1%)	0	10 (7%)	0.69
Tumor	7 (<1%)	0	7 (<1%)	0.33
HIV	2 (<1%)	0	2 (<1%)	0.28
**Disease severity**				0.001
Severe cases	705 (34%)	324 (52%)	381 (27%)	–
Non-severe cases	1,346 (66%)	299 (48%)	1,047 (73%)	–

BMI, body mass index; CVDs, cardiovascular diseases; DM, Diabetes Mellitus; CHD, Coronary heart disease; COPD, chronic obstructive pulmonary disease; CHF, Congestive heart failure; HIV, human immunodeficiency virus; COVID-19, coronavirus 2019; *p* values comparing survivors and deaths of patients are obtained from chi-square test, Fisher exact test, or Mann-Whitney *U* test. The significance threshold is at <0.05 and <0.01.

Briefly, of all 2,051 cases with confirmed COVID-19 illness, fever (1,658 of 2,051 cases; 81%), fatigue (1,420 of 2,051 cases; 69%) and dyspnea (1,109 of 2,051 cases; 54%) were the most frequent symptoms identified, followed by sore throat (974 of 2,051 cases; 47%), rhinorrhea (926 of 2,051 cases; 45%), cough (855 of 2,051 cases; 42%), sputum production (777 of 2,051 cases; 38%), chest pain (723 of 2,051 cases; 35%), and dizziness/headache (696 of 2,051 cases; 34%). The less common symptoms observed on admission were nausea/vomiting (554 of 2,051 cases; 27%), chill (433 of 2,051 cases; 21%), chest distress (416 of 2,051 cases; 20%) abdominal pain (366 of 2,051 cases; 18%) diarrhea (327 of 2,051 cases; 16%) and myalgia or arthralgia (301 of 2,051 cases; 15%). Among the overall population, a variety of coexisting illnesses were observed during the investigation. Hypertension (1,065 of 2,051 cases; 52%) and diabetes mellitus (1,010 of 2,051 cases; 49%) were the most common coexisting conditions identified among SARS-CoV-2 infected patients. The second most common associated comorbidities found in the study population were cerebrovascular disease (986 of 2,051 cases; 48%) and CHD (965 of 2,051 cases; 47%). The proportion of COPD, chronic renal disease, congestive heart failure, chronic liver disease, tuberculosis, asthma, and hepatitis B was 521 in 2,051(25%), 212 in 2,051(10%), 156 in 2,051 (8%), 96 of 2,051(5%), 88 of 2,051(4%), 55 of 2,051(3%), and 23 of 2,051(1%) respectively. Besides, the ratio of patients was less than 1% with a background marked by carcinoma, tumor, and HIV.

Compared with cases without cardiac injury, those who suffered from cardiac injury were more likely to be elderly [72.0 (65.0–92.5)] vs. [62.0 (55.5–76.0)] and male (69 vs. 62%). Dyspnea [(435 of 2,051 cases; 70%) vs. (674 of 2,051 cases; 47%)] and chest pain [(332 of 2,051 cases; 53%) vs. (391 of 2,051 cases; 27%)] were more likely to be present among them. Furthermore, almost all the associated comorbidities were present more often among cardiac injury patients except carcinoma, tumor, and HIV (Table 1). In terms of clinical classification, 705 (34%) patients were reported as severely ill and 1,346 (66%) patients were non-severe. Among severe cases, slightly more than half of the patients (324 of 623 cases; 52%) were identified with cardiac injury. During the diagnostic procedure, we found that 1,211 patients (59%) had a positive result in the first RT-PCR test, and 739 patients (36%) had a positive result in the second RT-PCR test. Surprisingly, another 101 patients (5%) remained negative until a third test.

### Radiologic and laboratory findings of hospitalized patients with COVID-19 illness

Table 2 summarizes the detailed results of radiological and laboratory outcomes of participants infected with SARS-CoV-2 on admission to the hospital. All participants underwent radiological evaluations. According to computed tomography and chest radiography findings, the proportion of bilateral pulmonary infiltration was seen in 76% of patients (1,643 of 2,051 cases), ground-glass opacity was shown in 31% of patients (633 of 2,051 cases), whereas, 26% showed consolidation (524 of 2,051 cases). Among cardiac injury patients, bilateral pulmonary infiltration (567 of 623 cases; 91%) was shown to be more common than in patients without cardiac injury (1,076 of 2,051; 75%). Moreover, the results of ground-glass opacity images were more often in patients with cardiac injury than compared to patients without cardiac injury. Among participants with cardiac injury, slightly more than half of patients 325 (52%) experienced examination of electrocardiogram (ECG) after they arrived in the hospital, and 212 of 325 ECGs (65%) were completed during the period with elevated cardiac biomarkers. Depression and inversion of T-wave, depression in ST-segment, and Q waves were detected in all two hundred and twelve ECGs, indicating myocardial ischemia.

**Table 2:** Radiologic and laboratory results of hospitalized patients with COVID-19 illness.

	**Patients No. (%)**			
		**Cardiac injury**		
	**All** ***n*** **(%)**	**With** ***n*** **(%)**	**Without** ***n*** **(%)**	***P*** **value**
**Radiological findings**				
Consolidation	524 (26%)	367 (59%)	157 (11%)	0.23
Ground-glass opacity	633 (31%)	512 (82%)	121 (8%)	0.04
Bilateral pulmonary infiltration	1,643 (80%)	567 (91%)	1,076 (75%)	0.001
**Laboratory findings**				
Neutrophilia (↑ neutrophil count)	739 (36%)	308 (49%)	(51%)	0.001
Leukocytosis (↑ WBC count)	780 (38%)	550 (88%)	(75%)	0.002
Thrombocytopenia (↓ platelets count)	1,028 (50%)	(57%)	(48%)	0.001
Lymphopenia (↓ lymphocytes count)	1,027 (50%)	568 (91%)	1,031 (72%)	<0.0001
**Elevation of cardiac injury biomarkers**				
NT-ProBNP > 600, pg/L	1,256 (61%)	268 (43%)	243 (17%)	0.002
Cardiac troponin I (above 99^th^ percentile)	513 (25%)	567 (91%)	1,171 (82%)	<0.0001
MYO, μg/L	353 (17%)	306 (49%)	430 (30%)	0.002
CK-MB >185, U/L	188 (9%)	132 (21%)	173 (12%)	0.001
LDH	841 (41%)	376 (60%)	501 (35%)	0.31
**Elevation of infection-related biomarkers**				
PCT > 0.5, ng/mL	534 (26%)	243 (39%)	291 (20%)	0.001
ESR > 20 mm/h	1,191 (58%)	369 (59%)	822 (57%)	0.16
IL-6 > 7 ng/L	1,047 (51%)	556 (89%)	1,161 (81%)	<0.0001
IL-8 > 62 ng/L	174 (8%)	151 (24%)	134 (9%)	0.051
IL-10	269 (13%)	388 (62%)	448 (31%)	0.071
TNF-α > 8.1 ng/L	971 (47%)	506 (81%)	(70%)	0.11
CRP > 10.0, mg/L	1,211 (59%)	575 (92%)	(81%)	0.001
Serum ferritin	1,192 (58%)	427 (68%)	765 (54%)	0.21
**Elevation of liver and renal biomarkers**				
ALT > 45, U/L	476 (23%)	155 (25%)	321 (22%)	0.26
AST > 45, U/L	417 (20%)	146 (23%)	271 (19%)	0.07
Albumin ≤ 35, g/L	841 (41%)	199 (32%)	642 (45%)	0.14
Serum creatinine > 1.3, mg/dL	475 (23%)	204 (32%)	271 (19%)	0.002
**Elevated Coagulation-related biomarkers**				
D-dimer > 1, μg/L	1,152 (56%)	526 (84%)	1,175 (82%)	0.001
Fibrinogen	1,249 (60%)	412 (66%)	1,131 (79%)	0.39

CK, creatinine kinase; ALT, alanine transaminase; AST, aspartate transaminase; PCT, procalcitonin; CRP, c-reactive protein; ESR, erythrocyte sedimentation rate; LDH, Lactate dehydrogenase; *p* values comparing survivors and deaths of patients are obtained from chi-square test, Fisher exact test, or Mann-Whitney *U* test, Lymphopenia was defined as a lymphocyte count of less than 1,500 per cubic millimeter. Thrombocytopenia was defined as a platelet count of less than 150,000 per cubic millimeter. The significance threshold is at <0.05 and <0.01.

On admission, the half proportion of the overall population had their platelet and lymphocyte count below the normal range and hence suffered from thrombocytopenia (1,028 of 2,051 cases) and lymphopenia (1,027 of 2,051 cases), respectively. Among 2,051 patients, more than 35% had an abnormality of neutrophils (739 of 2,051 cases) and WBC (780 of 2,051 cases) count. Cases with heart injury were more likely to experience abnormal levels of WBC (88 vs. 75%), platelets (57 vs. 48%), and lymphocyte (91 vs. 72%) count when compared with those without cardiac injury. Overall, among cardiac biomarkers, NT-proBNP was elevated in 61% (1,256 of 2,051), LDH in 41% (841 of 2,051 cases), cardiac troponin I in 25% (513 of 2,051 cases), MYO in 17% (353 of 2,051 cases), and CK-MB in 9% (188 of 2,051) of the whole population. In case of infection-related markers, CRP (59%; 1,211 of 2,051 cases), ESR (58%; 1,191 of 2,051 cases), IL-6 (51%; 1,047 of 2,051 cases) and serum ferritin (58%; 1,192 of 2,051 cases) were elevated in more than half of the population, followed by TNF-α (47%; 971 of 2,051 cases), PCT (26%; 534 of 2,051 cases), IL-10 (13%; 269 of 2,051 cases) and IL-8 (8%; 174 of 2,051 cases). In a substantial fraction of the infected individuals, coagulation markers such as d-dimer and fibrinogen were also elevated, with d-dimer in 56% and fibrinogen in 60% of the infected patients. Abnormal levels of liver and renal biomarkers were also reported among cardiac injury patients (except albumin; 32 vs. 45%) when compared to individuals without heart injury (ALT; 25 vs. 22%, AST; 23 vs. 19%, serum creatinine; 32 vs. 19%). On admission, infected participants with heart injury had an increased percentage of elevated levels of cardiac markers, inflammation, coagulation, and liver and renal contrasted with those without heart injury.

### Complications, treatment, and outcomes of hospitalized patients with COVID-19 illness

Table 3 summarizes the complications and therapies administered to COVID-19 participants while they were hospitalized. Throughout the follow-up period, the complications associated with COVID-19 disease were investigated. In aggregate, sepsis was the most prevalent condition among infected individuals. In addition, ARDS (33%; 677 of 2,051 cases) and respiratory failure (31%; 642 of 2,051 cases) occurred in more than 30% of all patients, of whom more than 60% were with cardiac injury. Other common complications occurred during hospitalization were septic shock (29%; 594 of 2,051 cases), bacteremia (17%; 349 of 2,051 cases), hyperglycemia (16%; 329 of 2,051 cases), electrolyte disturbance (11%; 226 of 2,051 cases), anemia (7%; 144 of 2,051 cases), hypoproteinemia (5%; 103 of 2,051 cases), coagulopathy (4%; 83 of 2,051 cases), acidosis (4%; 82 of 2,051 cases) and acute kidney injury (5%; 41 of 2,051 cases). Throughout the follow-up, an estimated 334 patients (16%) died, and 1,717 patients (84%) were recovered and discharged. Among 1,717 cases, 97% were without cardiac injury, and 52% were with cardiac injury. An elevated mortality rate (48%; 297 of 623 vs. 2%; 37 of 1,428) was observed in patients with heart injury as compared to those with no observed heart injury. During hospitalization pharmacological and non-pharmacological therapies were used to treat COVID-19, as appropriate. In pharmacological therapy, the ratio of antiviral therapy use was the maximum (94%; 1,932 of 2,051 cases), followed by intravenous corticosteroids (78%; 1,601 of 2,051 cases), intravenous immunoglobulin therapy (65%; 1,337 of 2,051 cases), and antibiotic therapy (65%; 1,338 of 2,051 cases). Forty-one patients (2%) were facilitated with continuous renal therapy among all participants. An aggregate of 1,871 patients was treated with oxygen support of whom high-flow nasal cannula oxygen therapy was given to 1,436 of 2,051 (70%) patients, non-invasive mechanical ventilation was provided to two-hundred and sixty-six patients (13%), and invasive ventilation was facilitated to one-hundred and sixty-nine patients (8%). Extracorporeal membrane oxygenation (ECMO) procedure was performed on twenty patients who were severely ill.

**Table 3:** Complications, treatment, and outcomes of infected patients during hospitalization.

	**Patients No. (%)**			
		**Cardiac injury**		
	**All** ***n*** **(%)**	**With** ***n*** **(%)**	**Without** ***n*** **(%)**	***P*** **value**
**Complications**				
Sepsis	1,271 (62%)	411 (66%)	860 (60%)	<0.0001
ARDS	677 (33%)	380 (61%)	297 (21%)	<0.0001
Respiratory failure	642 (31%)	388 (62%)	254 (18%)	0.001
Septic shock	594 (29%)	295 (47%)	299 (21%)	0.05
Bacteremia	349 (17%)	225 (36%)	124 (9%)	0.48
Hyperglycemia	329 (16%)	131 (21%)	198 (14%)	0.002
Electrolyte disturbance	226 (11%)	126 (20%)	100 (7%)	0.054
Anemia	144 (7%)	81 (13%)	63 (4%)	0.24
Hypoproteinemia	103 (5%)	51 (8%)	52 (4%)	0.63
Coagulopathy	83 (4%)	54 (9%)	29 (2%)	0.05
Acidosis	82 (4%)	27 (4%)	55 (4%)	0.17
Acute kidney injury	41 (2%)	19 (3%)	22 (1%)	0.09
**In-hospital treatment**				
Antibiotic therapy	1,337 (65%)	549 (88%)	788 (55%)	0.001
Antiviral therapy	1,932 (94%)	617 (99%)	1,315 (92%)	0.06
Intravenous corticosteroids	1,601 (78%)	561 (90%)	1,040 (73%)	0.001
High-flow nasal cannula oxygen therapy	1,436 (70%)	139 (22%)	1,297 (91%)	<0.0001
Non-invasive mechanical ventilation	266 (13%)	237 (38%)	29 (2%)	0.001
Invasive mechanical ventilation	169 (8%)	126 (20%)	43 (3%)	0.001
ECMO	21 (1%)	21 (3%)	0	0.91
CRRT	41 (2%)	39 (6%)	2 (0.14%)	0.39
Intravenous immunoglobin	1,338 (65%)	534 (86%)	804 (56%)	0.09
**Clinical outcomes**				
Recovered and discharged	1,717 (84%)	326 (52%)	1,391 (97%)	0.001
In-hospital death	334 (16%)	297 (48%)	37 (2%)	0.001

ARDS, Acute respiratory syndrome; ECMO, Extracorporeal membrane oxygenation; CRRT, Continuous renal replacement therapy. The significance threshold is at <0.05 and <0.01.

In comparison with those without heart injury, participants with heart injury were more likely to require non-invasive mechanical ventilation (38%; 237 of 623 vs. 2%; 29 of 1,428) and invasive mechanical ventilation (20%; 126 of 623 vs. 3%; 43 of 1,428). The use of pharmacological therapy such as an antibiotic (88 vs. 55%) and antiviral (99 vs. 92%) treatment, intravenous corticosteroids (90 vs. 73%), and immunoglobin (86 vs. 56%) was observed significantly greater in patients with heart injury against those without heart injury. Except for acidosis, the complications occurred more frequent in cardiac injury patients when compared with patients with no cardiac injury that included sepsis (66 vs. 60%), respiratory failure (62 vs. 18%), ARDS (61 vs. 21%), septic shock (47 vs. 21%), bacteremia (36 vs. 9%), hyperglycemia (21 vs. 14%), electrolyte disturbance (20 vs. 7%), anemia (13 vs. 4%), hypoproteinemia (8 vs. 4%), coagulopathy (9 vs. 2%), and acute kidney injury (3 vs. 1%).

### Potential risk factors and mortality in patients with cardiac injury

In univariable analysis, odds (OR) of in-hospital death were seen higher and significant in participants with elevated level of troponin I (OR: 23.09 with *p* < 0.0001), IL-6 (OR: 18.6 with *p* < 0.0001), and d-dimer (OR: 16.37 with *p* < 0.001), followed by age (OR: 2.21 with *p* < 0.0001), hypertension (OR: 4.35 with *p* < 0.001), CHD (OR: 3.39 with *p* < 0.001), lymphopenia (OR: 0.24 with *p* < 0.001), CK-MB (OR: 4.1 with *p* < 0.001), septicemia (OR: 11.67 with *p* < 0.001) and ARDS (OR: 7.07 with *p* < 0.001). Also, participants with heart injury showed a significant odd ratio of 8.42 linked with in-hospital mortality of infected individuals. After adjusting for age, hypertension, CHD, lymphocyte count, cardiac troponin I, CK-MB, IL-6, d-dimer, sepsis, and ARDS, the multivariate logistic regression model revealed a considerably higher risk of death than those who did not have a cardiac injury, Moreover, age (OR:1.99, *p* < 0.001), septicemia (OR:10.39, *p* < 0.001), and ARDS (OR:6.65, *p* < 0.0001) were all shown to be independent risk factors for COVID-19 patients' death. Under the multivariate odds ratio model, cardiac troponin I come up with an independent prognostic marker of mortality with an OR of 18.64 (*p* < 0.0001) (Table 4).

**Table 4:** Potential risk factors linked with in-hospital mortality of COVID-19 patients.

	**OR (95% CI)** **univariable**	***P*-value**	**OR (95% CI)** **multivariable**	***P* value**
Age (years)	2.21 (0.36–3.02)	**<0.0001**	1.99 (0.04–3.19)	**0.001**
Hypertension	4.35 (2.25–8.14)	**0.001**	3.88 (2.45–6.28)	0.32
DM	1.36 (0.13–7.17)	0.014	–	–
Cerebrovascular diseases	1.97 (1.01–4.69)	0.032	–	–
CHD	3.39 (2.12–6.52)	**0.001**	3.01 (2.50–5.37)	0.17
CHF	5.62 (4.19–11.23)	0.031	–	–
Asthma	1.96 (0.16–3.33)	0.062	–	–
Neutrophilia (↑ neutrophil count)	4.53 (3.33–10.14)	0.12	–	–
Leukocytosis (↑ WBC count)	0.64 (0.14–2.65)	0.008	–	–
Thrombocytopenia (↓ platelets count)	1.32 (0.42–2.07)	0.012	–	–
Lymphopenia (↓ lymphocytes count)	0.24 (0.09–2.45)	**0.001**	0.10 (0.04–2.02)	0.29
NT-ProBNP > 600, pg/L	2.47 (1.23–4.11)	0.002	–	–
Cardiac troponin I (above 99^th^ percentile)	23.09 (12.66–28.09)	**<0.0001**	18.64 (13.16–23.01)	**<0.0001**
MYO, μg/L	2.25 (0.31–3.47)	0.006	–	–
CK-MB >185, U/L	4.1 (2.24–7.13)	**0.001**	3.42 (1.14–6.07)	0.412
PCT > 0.5, ng/mL	11.36 (7.47–16.04)	0.009	–	–
IL-6 > 7 ng/L	18.36 (13.03–22.14)	**<0.0001**	14.57 (10.62–18.55)	0.071
CRP > 10.0, mg/L	6.65 (4.60–11.14)	0.014	–	–
Serum ferritin	0.78 (0.55–2.45)	0.047	–	–
D-dimer > 1, μg/L	16.37 (13.42–20.08)	**0.001**	13.65 (11.23–16.61)	0.55
Sepsis	11.67	**0.001**	10.39 (7.41–13.39)	**0.001**
ARDS	7.07	**0.001**	6.65 (4.04–8.91)	**<0.0001**
Respiratory failure	2.63	0.022	–	–
Cardiac injury	8.42	**<0.0001**	7.55 (4.08–10.11)	**0.001**
Hyperglycemia	5.25	0.012	–	–

OR, odd ratio; CI, confidence interval; DM, diabetes mellitus; CHD, coronary heart disease; CHF, chronic heart failure; MYO, myoglobin; CK-MB, creatinine kinase-myocardial band; PCT, procalcitonin; IL-6, interleukin 6; CRP, c-reactive protein; ARDS, acute respiratory distress syndrome. The bold shows significance at <0.01 and <0.05.

## Discussion

The present investigation indicated a statistically significant link between heart injury and death in corona-infected participants. Cardiac injury was shown to be related to an unexpectedly high incidence of death during hospitalization (30.4 %). The most common causes of COVID-19-induced mortality include sepsis and ARDS. In patients with heart impairment, sepsis and ARDS were revealed to be significant in multivariate analysis. Severe pneumonia has been linked to admittance to an intensive care unit (ICU), mechanical ventilation, and mortality in several epidemiological studies (4, 16). These findings imply that heart injury may be linked to COVID-19's poor clinical consequences. Consistently, the present analysis also identified 30.4% of participants with heart injury and showed that heart injury was linked independently with an elevated risk of death in corona-infected participants in the Pakistani setting. Acute sickness was more severe in people with heart injury than in those with no heart injury, as evidenced by abnormal radiological and laboratory findings including higher CRP, creatinine levels, and NT-proBNP; more bilateral pulmonary infiltration and multiple ground-glass opacity, and a significant portion necessitate invasive or non-invasive ventilation.

In an investigation of 121 SARS patients with cardiac abnormalities, hypertension was reported in 50.4% of those admitted to the hospital. Among them, 71.9% proved lasting tachycardia, with about 40% having constant tachycardia throughout outpatient follow-up (17). Although tachycardic cardiovascular abnormalities were prevalent in SARS individuals, they were typically self-limiting and not linked to a higher risk of mortality. In comparison to SARS, about 48% of participants with cardiac injury died in the hospital demonstrating that coronavirus-induced heart injury is significantly associated with serious clinical consequences in the present analysis. The etiology of cardiac injury in these COVID-19 individuals, however, is unknown.

Elevated hypersensitive troponin-I is a frequent co-morbidity in COVID-19 participants, with 12–77% reporting cardiac injury (18). COVID-19 has been implicated as a cause of myocardial damage, as evidenced by increased cardiac troponin-I levels (16, 19, 20). This study indicates that patients who have an increased cardiac troponin-I level during admission have a substantially greater in-hospital mortality rate than patients who do not have a cardiac injury. There are various possibilities for COVID-19-related myocardial damage, which is indicated by troponin-I increase (21, 22), which corroborates prior evidence linked to SARS and MERS outbreaks. Myocardial infarction, myocarditis, microangiopathy, and cytokine storm syndrome are manifestations of such pathways. Cytokine storm syndrome is a potentially fatal illness characterized by hemodynamic instability, systemic inflammation, multiple organ failure, and methemoglobinemia (23, 24). Cytokine storm syndrome is distinguished by an unregulated and defective immune response characterized by the continual activation and multiplication of macrophages and lymphocytes. In the current investigation, we also discovered that infection-related markers, for instance, PCT, CRP, and leukocyte count were considerably higher in individuals who had suffered heart injury. The triggering or increased production of these inflammatory cytokines can cause myocardial cell death or necrosis (25, 26).

Furthermore, patients with preexisting CVDs may be more vulnerable to coronavirus-induced heart injury in the current study, as approximately 65, 66, and 86% of participants with heart injury had a history of CHD, hypertension, and cerebrovascular disease, that were potentially more prevalent than in those with no cardiac injury (27–29). The outcomes of the present study corroborate with previous findings which have reported that COVID-19 participants who were severely ill had underlying cardiac abnormalities and elevated blood pressure (hypertension) (30, 31). Importantly, investigations have demonstrated that older patients with preexisting illnesses are more prone to be infected with SARS-CoV-2 with severe consequences, particularly those with a history of hypertension, CHD, and DM (32, 33). Even though there is insufficient evidence to conclude a direct connection between CV comorbidities and cardiac injury (34–36), it is plausible to think that cases with coronary artery disease (CAD) and heart failure (HF) are predisposed to heart injury and that once infected with severe respiratory illness (pneumonia), cardiac dysfunction or heart attack (myocardial ischemia) are more prone to occur, ultimately leads to unexpected deterioration. In the context of underlying cardiac events, acute inflammatory responses, on the other hand, might produce ischemia. During a systemic inflammatory response, the infection within coronary atherosclerotic plaques becomes more intensified, making them more susceptible to rupture (37, 38). Inflammation also promotes endothelial dysfunction and increases blood procoagulant activity, which may result in an occlusive thrombus forming over a ruptured arterial plaque (39, 40). Based on the research findings, we believe that a high inflammatory response combined with underlying CVD may be the cause of the heart injury found in corona-infected individuals.

## Conclusion

Cardiac injury is a frequent complication in corona-infected participants and is found associated with an elevated risk of in-hospital death. Though the specific mechanism of heart injury necessitates an additional investigation, the statistics provided here underscore the necessity to include this complication in the treatment and management of corona-infected patients.

## Data availability statement

The raw data supporting the conclusions of this article will be made available by the authors, without undue reservation.

## Ethics statement

The studies involving human participants were reviewed and approved by Ministry of Health of Pakistan, granted clearance for the study (KIIT 2022/PK 2022-104-MS 68). The Ethics Committee waived the requirement of written informed consent for participation. Written informed consent was not obtained from the individual(s) for the publication of any potentially identifiable images or data included in this article.

## Author contributions

All authors listed have made a substantial, direct, and intellectual contribution to the work and approved it for publication.

## Conflict of interest

The authors declare that the research was conducted in the absence of any commercial or financial relationships that could be construed as a potential conflict of interest.

## Publisher's note

All claims expressed in this article are solely those of the authors and do not necessarily represent those of their affiliated organizations, or those of the publisher, the editors and the reviewers. Any product that may be evaluated in this article, or claim that may be made by its manufacturer, is not guaranteed or endorsed by the publisher.
